# Order enables efficient electron-hole separation at an organic heterojunction with a small energy loss

**DOI:** 10.1038/s41467-017-02457-5

**Published:** 2018-01-18

**Authors:** S. Matthew Menke, Alexandre Cheminal, Patrick Conaghan, Niva A. Ran, Neil C. Greehnam, Guillermo C. Bazan, Thuc-Quyen Nguyen, Akshay Rao, Richard H. Friend

**Affiliations:** 1 Department of Physics, Cavendish Laboratory, University of Cambridge, 19 JJ Thompson Avenue, Cambridge, CB3 0HE UK; 2 Center for Polymers and Organic Solids, University of California, Santa Barbara, Santa Barbara, CA 93106 USA

## Abstract

Donor–acceptor organic solar cells often show low open-circuit voltages (*V*_OC_) relative to their optical energy gap (*E*_g_) that limit power conversion efficiencies to ~12%. This energy loss is partly attributed to the offset between *E*_g_ and that of intermolecular charge transfer (CT) states at the donor–acceptor interface. Here we study charge generation occurring in PIPCP:PC_61_BM, a system with a very low driving energy for initial charge separation (*E*_g_−*E*_CT_ ~ 50 meV) and a high internal quantum efficiency (*η*_IQE_ ~ 80%). We track the strength of the electric field generated between the separating electron-hole pair by following the transient electroabsorption optical response, and find that while localised CT states are formed rapidly (<100 fs) after photoexcitation, free charges are not generated until 5 ps after photogeneration. In PIPCP:PC_61_BM, electronic disorder is low (Urbach energy <27 meV) and we consider that free charge separation is able to outcompete trap-assisted non-radiative recombination of the CT state.

## Abstract (web summary)

Achieving charge separation in low energy loss organic heterojunctions is crucial to the efficiency of donor-acceptor solar cells, whilst the timescale of the process remains largely unknown. Here, Menke et al. observe slow charge separation up to 5 ps in a system with small energy offset of 50 meV.

## Introduction

Organic heterojunctions currently require an electron donor–acceptor (DA) interface to separate molecular excitons into free charge carriers^1^. It has been considered that an offset in molecular orbital energy levels at the DA interface is required in order to make electron-hole separation possible^2–4^. In fullerene-based organic solar cells, for example, this offset of ~300 meV allows electrons to be injected from the donor into a set of delocalised states within a fullerene aggregate, resulting in ballistic electron-hole separation in under 100 fs^5,6^. This fast timescale ensures that free charge carrier generation is nearly 100% efficient. Smaller orbital offsets are required, however, to reduce photovoltage loss in organic solar cells^7^ (OSCs) and there is less understanding how smaller offsets may affect this separation timescale.

Many factors have been considered to influence electron-hole separation at organic heterojunctions^8,9^. Recent studies have drawn connections with the DA molecular conformation^10–12^, vibrational coherence^13^, molecular orbital energy levels^14^, presence of fullerene aggregates^15^, dielectric constant^2^ and local mobility^16^ among other properties. At the heart of these analyses is how charges overcome the binding energy of the geminate interfacial state. The notion of a minimum offset thus emerges from an enthalpic perspective of charge separation. If one also considers the gain in entropy from the increase in possible number of states, it follows that charge separation can proceed even in the absence of an enthalpic driving force^17^. In practice, we know this situation must be true as there are now reports of OSCs with high internal quantum efficiencies for charge generation with offset energies <100 meV^18–20^. The timescale and dynamics of electron-hole separation in these systems, however, have remained unclear.

**Fig. 1:**
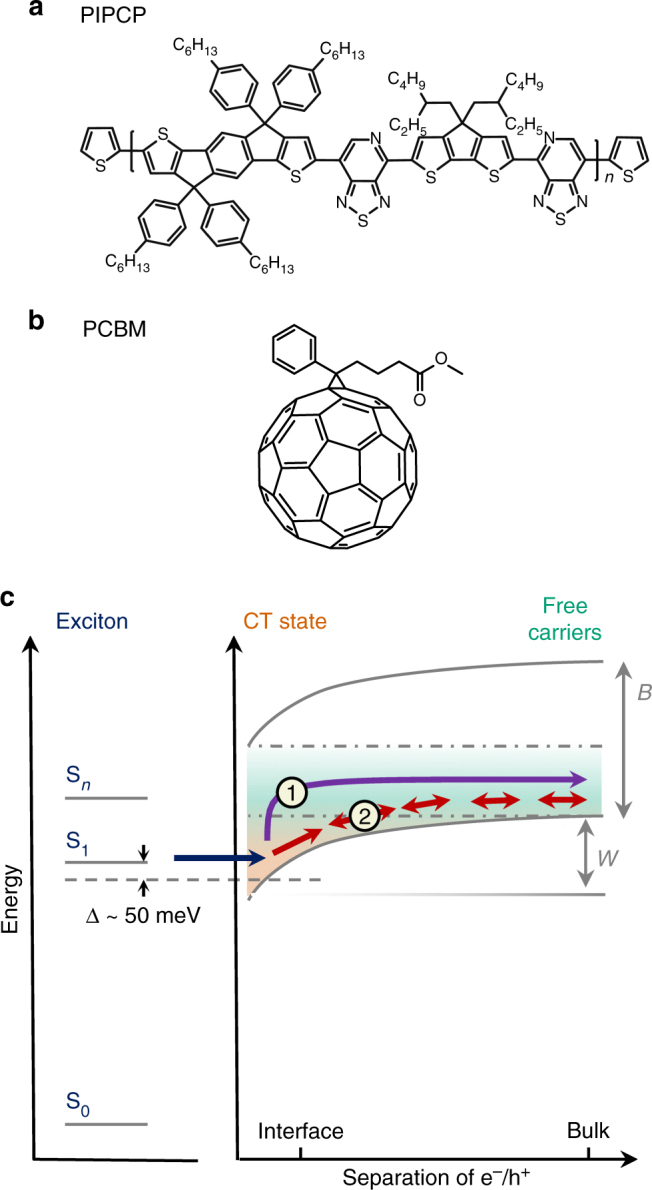
Charge separation in low-energy loss heterojunctions. Molecular structures for PIPCP **a** and PCBM **b**. **c** Schematic state diagram for electron-hole separation in an organic heterojunction with a small offset energy between the singlet exciton (S_1_) and the geminate charge transfer (CT) state (dashed line). In contrast to systems with larger-offset energies, direct access to delocalised states, with bandwidth *B*, is not energetically possible from S_1_. As a consequence, charge separation must overcome the Coulomb potential (*W*) by either thermal population into delocalised states (1) or successive hopping (2) within the density of states. Note that orange represent CT states at the donor–acceptor interface and green represents delocalised, free carriers

Here, we probe the dynamics of the initial electron-hole separation for an organic heterojunction based on the donor polymer PIPCP and the acceptor [6,6]-phenyl-C_61_-butyric acid methyl ester (PCBM) (Fig. 1a, b)^18,21^. This system is of particular interest because there is only a ~50 meV difference between the optical energy gap (*E*_g_) and the energy of the interfacial CT state (*E*_CT_) as determined by complementary, sensitive measurements of absorption and electroluminescence^18^. We define the offset energy as the difference between *E*_g_ and *E*_CT_. Despite this small offset energy, OSCs fabricated from blends of PIPCP and PCBM exhibit large photocurrents and high internal quantum efficiencies (*η*_IQE_ > 80%). Importantly, this blend is notable for having a very small degree of electronic disorder (Urbach energy ~27 meV)^18^. As the offset energy is less than the Coulomb binding energy of the geminate CT state, direct coupling to delocalised states is not possible (Fig. 1c). Instead, the geminate CT state either waits until it can be excited upward into a set of delocalised states or undergoes incoherent hopping within the density of states, provided there is a sufficient entropic gain^22^.

To probe electron-hole separation in PIPCP:PCBM blends, we utilise a pump-push-probe (PPP) transient absorption (TA) spectroscopic technique. In contrast to two-pulse, pump-probe TA spectroscopy, PPP records the differential TA under the presence of an infrared push pulse selected to interact with photogenerated excited states^23^. This technique has been recently used to reveal the electroabsorption (EA) signature of molecules near the DA interface in the presence of the dipolar electric field induced by electron-hole separation^24^. We are able to use this differential EA signature to extract the energy stored in the electric field as a function of time after initial photogeneration. We find that the maximum energy stored in the electric field takes ~5 ps, 10 times slower than previously studied polymer-fullerene and non-fullerene organic heterojunctions. We attribute the ability to generate charges slowly yet efficiently to the remarkably low disorder in the system and present a conceptual model invoking the Urbach tail in the density of states that can inform us of the upper bound for the electronic disorder tolerable to achieve efficient carrier separation in systems with small offset energies.

## Results

### Pump-probe transient absorption

Previous studies have rigorously analysed the steady-state absorption, luminescence and >10 ps TA profiles for PIPCP:PCBM blends^18,21,25^. To unravel the charge generation dynamics on the sub-10 ps timescale, we employ an ultrafast, broadband pump-probe (PP) TA spectrometer capable of providing <15 fs, 750 nm-centred pump pulses, leading mainly to excitation of PIPCP in the blend. It allows us to probe the change in absorption (Δ*T*/*T*) as a function of pump-probe delay from *λ* = 550–1350 nm with <15 fs time resolution^26^.

**Fig. 2:**
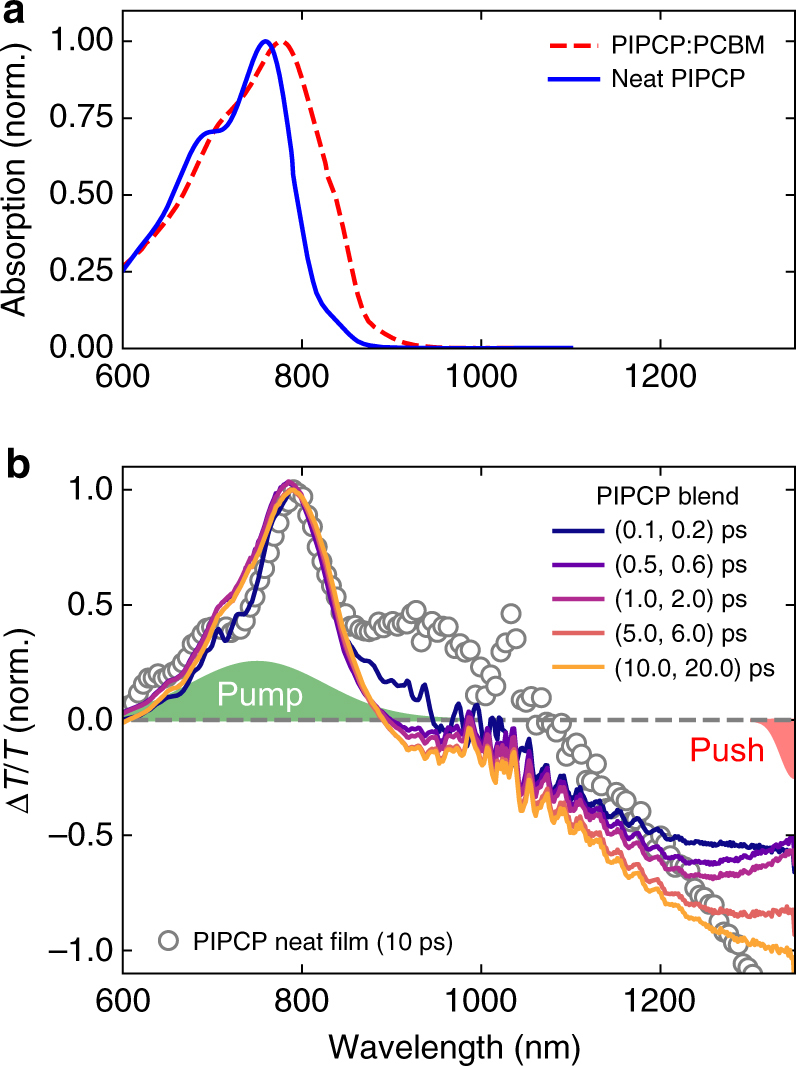
Transient optical absorption of PIPCP:PCBM. **a** Steady-state absorption for neat PIPCP and PIPCP:PCBM blend. **b** PP transient absorption spectra of the PIPCP:PCBM blend at selected pump-probe delays. The TA spectrum of neat PIPCP at 10 ps is shown in grey circles for comparison. Spectra were recorded at an excitation fluence of 50 μJ cm^−2^. Noted are the approximate spectra for the pump and push pulses. Non-normalised data can be found in the Supplementary Fig. 1

Figure 2a shows the steady-state absorption for neat PIPCP and a PIPCP:PCBM blend, and Fig. 2b shows the TA spectra for a blend and a neat film. Non-normalised TA spectra are shown in Supplementary Fig. 1. The neat TA spectra show the initial excitation of PIPCP with ground-state bleaching located at *λ* = 800 nm. The corresponding stimulated emission (SE) at *λ* = 950 nm and photoinduced absorption (PIA) at *λ* = 1350 nm indicate the photogeneration of PIPCP excitons with a lifetime of 30 ps, in agreement with previous measurements^25^. In the blend, the PIA and SE characteristics of the PIPCP excitation are rapidly quenched (250 fs) via charge transfer to PCBM giving rise to the formation of PIPCP cation absorption at *λ* = 1250 nm. The PCBM anion absorption is not observed due to its low absorption cross section and absence of sharp features. On the 10 ps timescale, a PIA located at *λ* = 1350 nm is observed, which has been previously identified as PIPCP triplet excitons^25^. We point out that the appearance of triplet excitons is the result of very rapid non-geminate recombination at the high-charge densities generated during the experiment.

### Pump-push-probe transient absorption

**Fig. 3:**
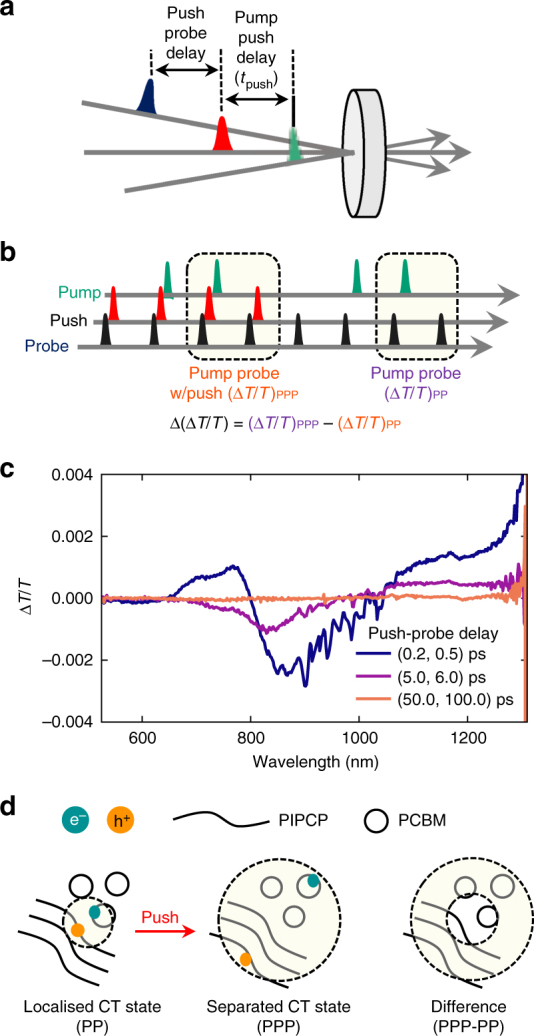
Pump-push-probe isolates electroabsorption. **a** Schematic for the time relationship between the pump, push and probe pulses. **b** Pulse sequence used to record the TA (∆*T*/*T*) with and without the push pulse where their difference is the push-induced change in TA (∆(∆*T*/*T*)). **c** Push-induced changes in TA spectra for a pump-push (*t*_push_) delay of 1.2 ps at various push-probe delays at a pump fluence of 50 μJ cm^−2^ and push fluence of 90 μJ cm^−2^. **d** Schematic describing how the push pulse interacts with the localised CT states resulting in a more separated CT state. The shaded region reflects the region affected by the dipolar electric field generated from the electron-hole pair. Differential, push-induced TA reflects the additional electroabsorption generated by the push pulse where larger signals correspond to more initially localised CT states

To investigate the nature of interfacial CT states, we make use of a third, push pulse to provide extra energy to these excited states and observe their relaxation (Fig. 3a). The push pulse is tuned at *λ*_push_ = 1350 nm to be resonant with the red edge of the PIPCP cation. In this experiment, the push pulse is triggering an electronic transition from a given excited electronic state to a higher-lying electronic state. Sequentially varying the combinations of pump, push and probe pulses allows the measurement of pump-probe TA, push-probe TA and pump-push-probe TA (Fig. 3b). The push-induced change in pump-probe TA is defined as the difference between the pump-push-probe and the pump-probe combinations and results in a differential TA signal (Δ(Δ*T*/*T*)) (Supplementary Fig. 2). The push pulse intensity is sufficient to excite ~1% of the pump-generated excited electronic states, but low enough to prevent multiphoton electronic excitation of the ground state. This can be controlled by monitoring the push-probe TA signals. The absence of multiphoton excitation (Supplementary Fig. 3) of the ground state guarantees that the push pulse only interacts with excited states, and therefore that the number of electronically excited molecules is unchanged. The push pulse can be delayed with respect to the pump pulse so as to excite different electronic excited states as their population evolves in time. The PPP signal is the push-induced change in the PP TA. It monitors the temporal response of the out-of-equilibrium states originally generated by the pump that interact with the push.

Figure 3c shows differential TA spectra for a PIPCP:PCBM blend for a pump-push delay (*t*_push_) of *t*_push_ = 1.2 ps at various push-probe delays at a pump fluence of 50 μJ cm^−2^ and push fluence of 90 μJ cm^−2^. The push-induced response starts at the push arrival time. We observe bleaching of the near infrared PIAs through the instrument-limited rise of positive signals in the *λ* = 1100–1350 nm region (maximum at the edge of the spectral window with a shoulder at *λ* = 1160 nm). This indicates push-induced depletion of pump-induced states and signals that we are interacting with species, which have PIA in this spectral region (eg, CT states and charges). The initial push response also contains a derivative-like feature (*λ* = 550–1000 nm) with positive and negative lobes and subtle vibronic structure located in the region of PIPCP blend absorption. They are rapidly decaying on the ps timescale and lead to the broadening and blue-shifting of the negative feature while the PIA depletion signal is decaying. No signal lasting >100 ps is recorded, signalling that the system is able to fully recover to the unpushed state by 100 ps. Note that the same features were identified at low fluences down to 2 μJ cm^−2^ for the pump and 9 μJ cm^−2^ for the push.

**Fig. 4:**
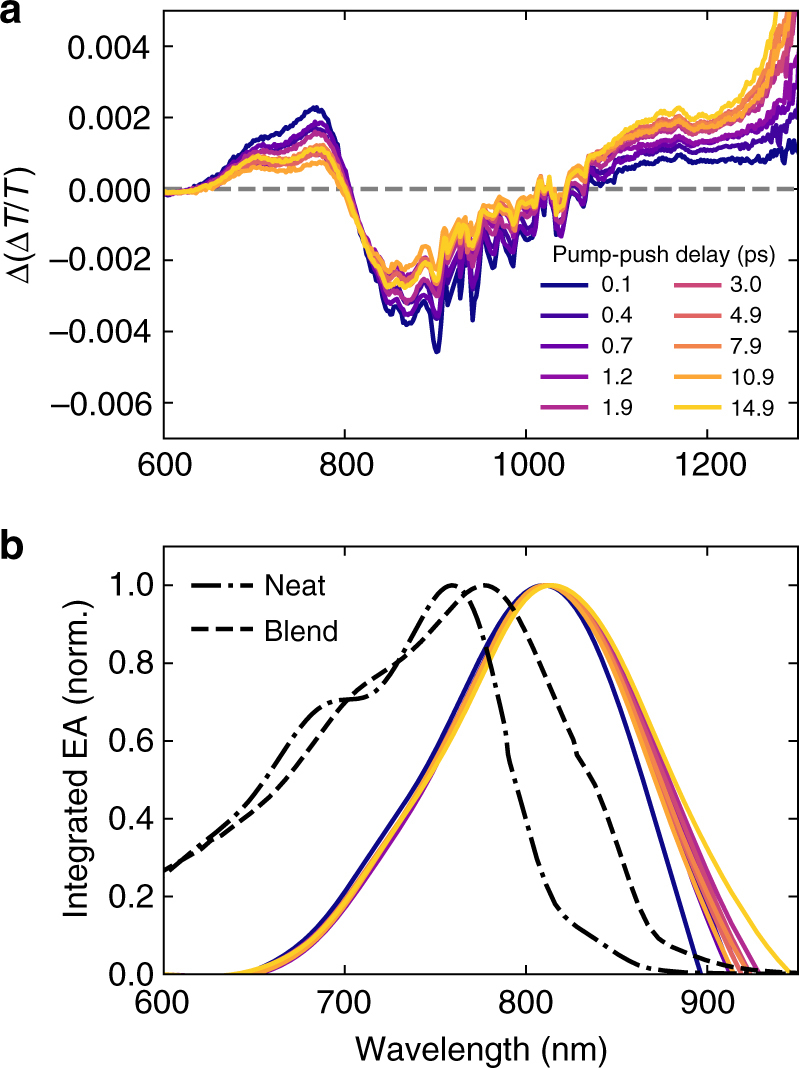
Evolution of push-induced electroabsorption reveals ordered interface. **a** Push-induced, differential TA spectra averaged between 0.25 and 0.50 ps push-probe delay at various pump-push delays at a pump fluence of 50 μJ cm^−2^ and push fluence of 90 μJ cm^−2^. **b** Integrated push-induced, differential TA spectra from **a** as compared to the neat and blend steady-state absorption for PIPCP

Recalling the photophysical scheme presented in Fig. 1c and the decay of the SE of singlet excitons in Fig. 2b, we can confirm that PIPCP singlet excitons are rapidly quenched by charge transfer to fullerenes after excitation. The resulting CT states are localised at the DA interface until they can overcome by thermal excitation their mutual Coulomb attraction and separate to form separated, free charges. To avoid excitation of singlet and triplet excitons, we investigated pump-push delay times of *t*_push_ = 0.1–15 ps—after singlet excitons are dissociated but before triplet excitons are formed via non-geminate recombination (Fig. 4a). In comparing the push-induced differential TA for push-probe delays between 0.25 and 0.50 ps, all pump-push delays investigated share two features. First, we attribute the differential response at *λ* = 600–1000 nm to EA, which decays on a 10 ps timescale. Second, we attribute the PIA bleach at *λ* > 1000, which decays on a 1 ps timescale to pushed CT states and pushed charges (Supplementary Fig. 4).

### Electroabsorption at the buried interface

The low-energy push pulse excites both localised CT states and separated charges, consistent with previous measurements by Jakowetz et al^24^. In the case of localised CT states, the push pulse can provide them with sufficient energy to overcome their mutual Coulomb attraction and separate (Fig. 3d). Push-induced EA is generated due to the change in electric field generated by the separating charges. The change in electric field induces a Stark shift in the absorption spectrum of nearby molecules and inserts a derivative-like feature into the TA due to the corresponding shift in the transmission of the sample. Note that the EA signal (*λ* < 1000 nm) lives 10 times longer than the PIA bleach (*λ* > 1000 nm) signalling that even after pushed hole polarons (from both CT states and separated charges) on PIPCP have thermalised, there is still push-induced EA resulting from the further separated electron-hole pair (Supplementary Figs. 5 and 6).

It is important to note that in the case of separated charges the push pulse is not expected to further increase the average separation. Therefore, no push-induced EA signal is expected for separated carriers as there is no net change in the electric field. For localised CT states, the push-induced EA signal, in contrast, is strong. In this way, we can simply consider that push-induced signals at *λ* < 1000 nm are due to CT states, which have separated due to the push pulse, whereas push-induced signals at *λ* > 1000 nm provide a measure of the amount of pushed species (CT states and free charges).

With an understanding of the push-induced photophysics in PIPCP:PCBM blends, the EA signature can be assessed. Figure 4a shows the push-induced, differential TA between 0.25 and 0.50 ps after the push pulse as a function of pump-push delay. Using PPP, we are able to isolate the push-induced EA response. A large value shows that most excitations are localised and a small value signals that CT states have undergone separation to form free charges. The strength of the signal is also proportional to the number of pushed charges. In Fig. 4a, we observe both a systematic decrease in the EA signal (*λ* = 800–850 nm) as well as an increase in CT state plus charge bleach (*λ* = 1000–1350 nm). This indicates that the strength of the EA signal on a per pushed-charge basis is decreasing with increasing *t*_push_. This is consistent with CT states that are separating.

The spectral shape of the EA response is determined by the spectral shape of the molecular absorption profile. In line with Jakowetz et al.^24^, integration of the EA as a function of pump-push delay time can be used as a local probe of DA interfacial morphology, as the EA generated during electron-hole separation predominantly affect molecules near this separation process. Figure 4b shows the integrated curves from Fig. 4a as compared to the steady-state blend absorption measured by photothermal deflection spectroscopy.

We observe that the peak shifts very little on these timescales, in contrast to previous reports for other polymer-fullerene blends^24^. In PCDTBT:mPCBM, for example, the EA-extracted absorption initially blue-shifts on the 100 fs timescale then subsequently red-shifts on the 10 ps timescale. This is interpreted as separated charges delocalising into more ordered regions before downhill relaxation to lower-energy, disordered regions of the blend^24^. In PIPCP:PCBM, the lack of a shift in the EA-extracted absorption may signal that the blend is highly ordered near the DA interface (recalling that the bulk blend absorption is very well-ordered). We are able to confirm that the low Urbach energy measured for the bulk blend indeed translates to low disorder on the nanoscale at and around the DA interface.

The inferred absorption of the interfacial states remains nearly constant at 1.51 eV and is ~80 meV red-shifted as compared to the bulk blend absorption. This energy is in agreement with the CT energy predicted from time-dependent density functional theory of 1.51 eV^25^. If there was a high degree of domain purity at the DA interface, we would expect the EA response to resemble the absorption of pure PIPCP peaked at 1.6 eV. Instead, the lower-energy absorption captured from the push-induced EA strongly suggests that the majority of excited states for PIPCP near the DA interface have a stronger degree of CT character. As the integrated EA is red-shifted from the blend, we conclude that the interfacial regions are of lower energy than that of the bulk film and that charges remain in these regions during separation. This might be the case if, for instance, charge separation is mainly occurring by hole polarons moving along the PIPCP backbone.

**Fig. 5:**
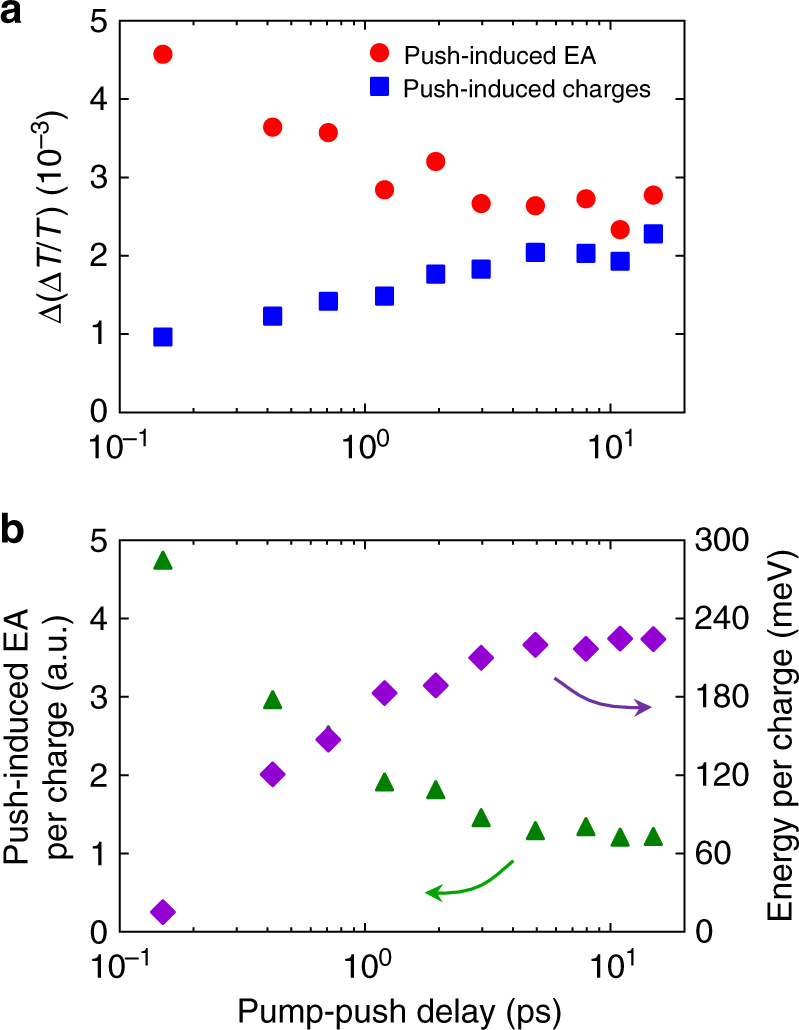
Timescale for charge separation in PIPCP:PCBM. **a** Differential TA signal recorded between 0.25 and 0.50 ps after the push pulse as a function of pump-push delay for the push-induced EA (*λ* ~ 850 nm) and the push-induced charges (*λ* = 1150–1200 nm). Note that the latter includes both CT states and separated charges. **b** Push-induced EA on a per charge basis and the energy per charge stored in the dipolar electric field resulting from CT state separation as a function of pump-push delay

Inspection of Fig. 4a also reveals that the amplitude of the differential EA response relative to the PIA bleach is a function of the pump-push delay time. Figure 5a shows the absolute value of the push-induced EA signal near *λ* = 850 nm and the maximum of the push-induced charge carrier signal between *λ* = 1150 and 1200 nm as a function of pump-push delay time. Note that the charge carrier signal will be composed of both CT states and separated charges. The intensity of push-induced charge carriers changes by a factor of ~2, while the push-induced EA signal decreases by a factor of ~2. This behaviour is expected if the CT states are indeed separating and the proportion of separated carriers, which will not produce a differential EA response, is increasing relative to the proportion of CT states.

## Discussion

Figure 5b shows the push-induced, differential EA normalised to the charge carrier population (CT states plus separated charges). As this signal plateaus on the 1–5 ps timescale, we infer that the timescale for CT state separation is between 1 and 5 ps for PIPCP:PCBM. As we develop in the Supplementary Note 1, we can estimate the pump-induced EA signal from the push-induced differential EA signal. We have calibrated the intensity of the EA response using quasi-steady-state EA measurements using an externally applied electric field. Note that this calibration assumes that the orientation of the DA interface is random with respect to the electrodes (for a detailed description, see the Supplementary Information of ref. ^2^). For this reason, we provide an estimate for the energy stored in the dipolar electric field generated after CT state separation. This value should be similar to the Coulomb binding energy for a CT state. Figure 5b shows this energy on a per charge basis as a function of pump-push delay. We find that the stored energy plateaus to a value of ~210 meV on the same timescale as CT state separation. This timescale is ~10 times slower that what is observed in other fullerene blends such as PCDTBT:PCBM (<100 fs)^5^, PTB7-Th:PCBM (<100 fs) and other non-fullerene blends such as PTB7-Th:bay-di-PDI (<100 fs). Note all these systems have offset energies >200 meV. While the timescale for PIPCP is ~10 times slower, charge generation can still be efficient, provided this process is faster than geminate CT state decay pathways, which typically have been found to occur on the 100 ps–1 ns timescale.

**Fig. 6:**
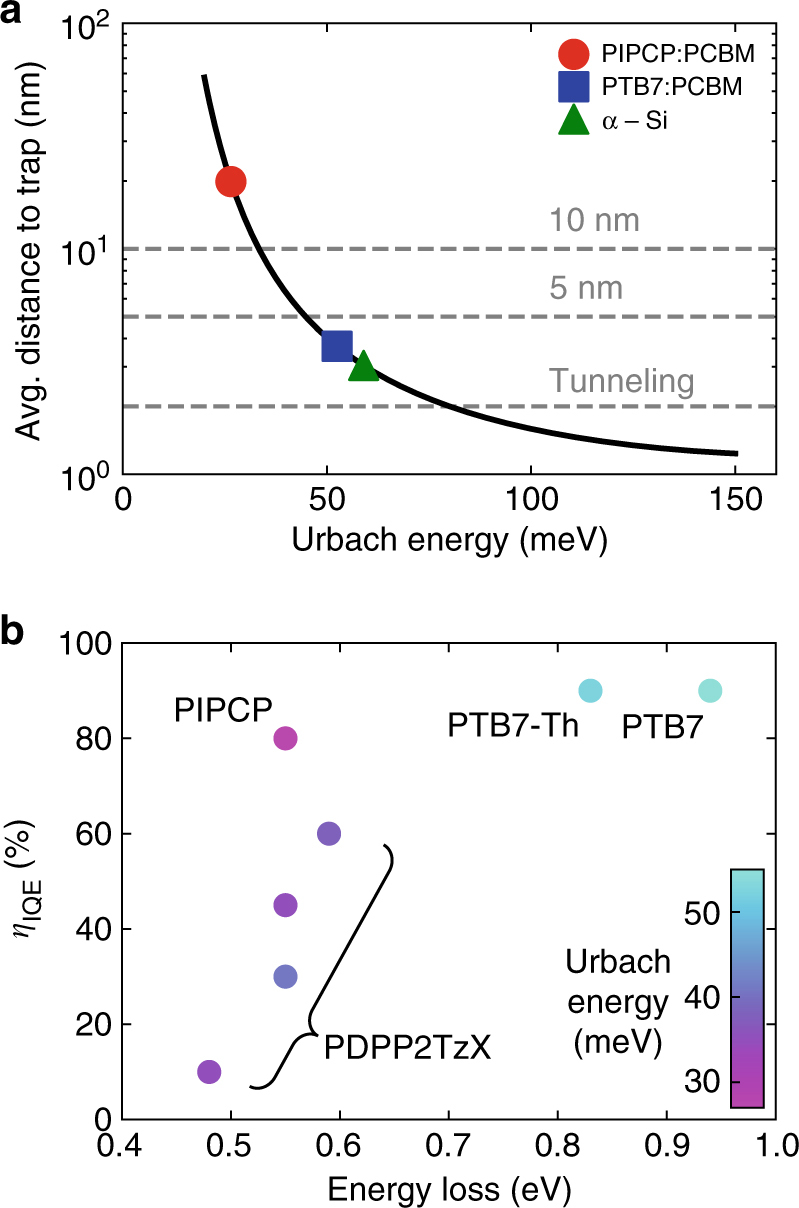
Low Urbach energy is necessary for large internal quantum efficiencies. **a** Estimation for the average distance to CT-state trap sites as a function of Urbach energy. Here, we define a trap site as any site with an energy >25 meV<*E*_g_−0.1 eV. **b** *η*_IQE_ vs energy loss (*E*_g_ − *V*_OC_) for a variety of polymer-fullerene blends. The colour of the marker denotes its Urbach energy. For the PDPP series, X is T, 2T, BDT and DTP^29^

It is important to note that this relatively long timescale for electron-hole separation can allow localised CT states to migrate towards or tunnel to lower-energy trap sites in their density of states. If this were to happen, it is likely that the binding energy may increase, ie, the electron and hole may come closer together, precluding efficient electron-hole separation. The number of low-energy sites is related to the level of disorder at the DA interface. For PIPCP blends, the low degree of electronic disorder allows geminate CT states to stay at a high enough energy for long enough to enable efficient electron-hole separation. To demonstrate this relationship, we provide an estimate for the average spacing between CT states with the Urbach tail density of states. To do this, we choose a simple model in which a CT state below an empirical site energy cutoff (the disorder-free energy gap) is no longer able to further separate (Supplementary Fig. 7). This would manifest as a reduction in the internal quantum efficiency. As has been empirically found for a broad array of both organic and inorganic semiconductors, we assume an exponential density of states near the energy gap where the Urbach energy determines the degree of disorder. For a given cutoff energy, here selected to be 0.1 eV below the top of the Urbach tail, we can integrate to find an estimate for the number of low-energy, trap states and their corresponding average separation as a function of Urbach energy. Figure 6a displays the result of this simple, model calculation and highlights representative locations for PIPCP:PCBM, PTB7:PCBM and ɑ-Si.

As the Urbach energy increases, the average distance to a trap site is reduced. It is how this length-scale changes with Urbach energy, which we find is critical. Previous reports suggest the diffusion length for interfacial CT states to be ~5 nm^27^, and we consider that tunnelling between CT states is expected to turn on for separations <2 nm. For systems with an Urbach energy >~40 meV, consequently, our model would predict a low internal quantum efficiency since geminate CT states can quickly find a low-energy site from which separation can no longer occur. In the case of PTB7:PCBM and PTB7-Th:PCBM, however, this behaviour is not observed since PTB7 and PTB7-Th blends have a large offset energy (~250 meV)^28^ allowing electrons to be directly injected into delocalised accepting states within a fullerene cluster. For systems with small offset energies designed to have minimal energy loss, direct coupling to these states is unlikely. Our model predicts that the DA blend should have an Urbach energy of 30 meV or less in order for charge separation to be efficient with a small DA offset energy and where the timescale for charge separation is >1 ps.

To explore this hypothesis, Fig. 6b shows the *η*_IQE_ vs energy loss for a PIPCP, PTB7, PTB7-Th and a series of diketopyrrolopyrrole-based copolymers reported by Li et al^29^. The *η*_IQE_ was measured and found to be 80% (Supplementary Fig. 8). Note that for consistency, we have chosen to plot the *η*_IQE_ as a function of energy loss. While the total energy loss will have contributions from both the offset energy as well as recombination, recombination energy loss has been found to almost always fall between 0.5 and 0.6 eV^30^. PTB7 and PTB7-Th are notable for having high *η*_IQE_, yet we can see that they likely require these large offset energies to overcome the disordered nature of the blend. The PDPP series of polymers was synthesised in order to begin understanding the nature of charge generation as a function of offset energy. From this initial study, one might have concluded that a certain offset energy is required. Rather, our model suggests that the PDPP series may have been too disordered, so that localised CT states were becoming trapped before charge separation could occur.

In this work, we have shown that charge separation for localised CT states in PIPCP:PCBM, a low-offset-energy polymer-fullerene heterojunction, is efficient despite a slower separation timescale (up to 5 ps) than that typically observed in larger-offset heterojunctions (100 fs). This process can remain efficient when low energetic disorder of the blend is sufficiently low to allow for an extended amount of time before CT states become trapped in the tail of their density of states. Consequently, focus should be placed on identifying systems with low Urbach energies, to develop high-efficiency blends with low-offset energies. Within this simple model, we propose that blends should exhibit Urbach energies <30 meV.

## Methods

### Materials and thin-film fabrication

PIPCP (*M*_n_ = 66 KDa, PDI = 1.6) and PDPP2TzX (X = T, 2T, BDT, DTP) polymers were synthesised as reported elsewhere^21,29^. PC_61_BM was used as obtained from Solenne B.V. PIPCP:PC_61_BM (1:2, w/w) blend and pristine PIPCP solutions were prepared in a mixed solvent of chloroform and chlorobenzene (6:4, v/v) into a 20 mg/ml concentration and left for stirring overnight. PDPP2TzT:PC_61_BM and PDPP2TzBDT:PC_61_BM (1:2, w/w) were prepared in chloroform with 10 vol.% o-dichlorobenzene (o-DCB). PDPP2Tz2T:PC_61_BM and PDPP2TzDTP:PC_61_BM (1:3, w/w) were prepared in chloroform with 5 vol.% o-DCB. Quartz substrates were cleaned by sonicating in tergitol, deionised water, acetone and isopropanol for 5 min successively, followed by oxygen plasma treatment for 10 min. Thin films were spin-coated in a N_2_-filled glove box.

### Transient absorption spectroscopy

The TA spectrometer used for these experiments is described elsewhere^26^, the pump-push-probe capability is described in detail in the Supplementary Note 2. In brief, the setup is based on a PHAROS (Light Conversion). The push pulse is formed from seeding a commercial OPA (ORPHEUS, Light Conversion). A home built non-collinear parametric amplifier is used for the pump pulses. The probe pulse is a white light continuum generated in a YAG crystal.

### Data availability

The TA data that support the findings of this study are available in the Cambridge Apollo Data Repository with the identifier 10.17863/CAM.15808.

## Electronic supplementary material

Supplementary Information
