# Supplementary material for: Order enables efficient electron-hole separation at an organic heterojunction with a small energy loss
Source: Nat Commun. 2018 Jan 18;9:277. doi: 10.1038/s41467-017-02457-5 (PMC5773693; doi:10.1038/s41467-017-02457-5)
Supplement: Supplementary file 1 — Supplementary Information [file 41467_2017_2457_MOESM1_ESM.pdf]

1. Pump-Probe Transient Absorption for PIPCP:PCBM

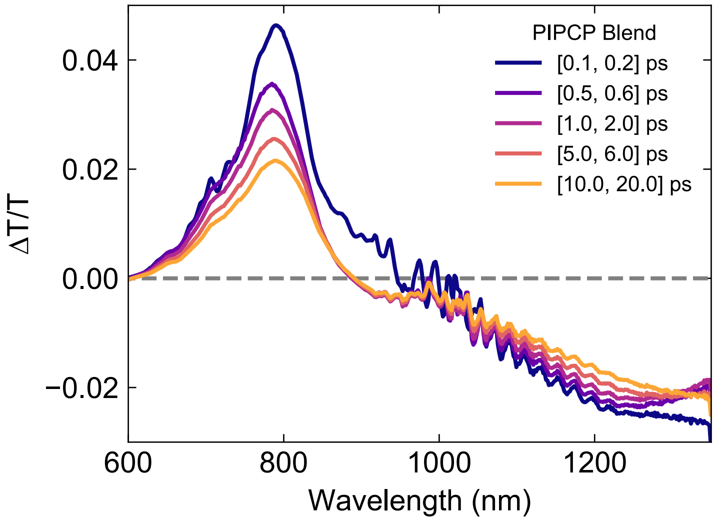

Supplementary Figure 1 | Non-normalized pump-probe transient absorption (ΔT/T) from Fig. 2b in the manuscript.

2. Pump-Push-Probe Transient Absorption for PIPCP:PCBM

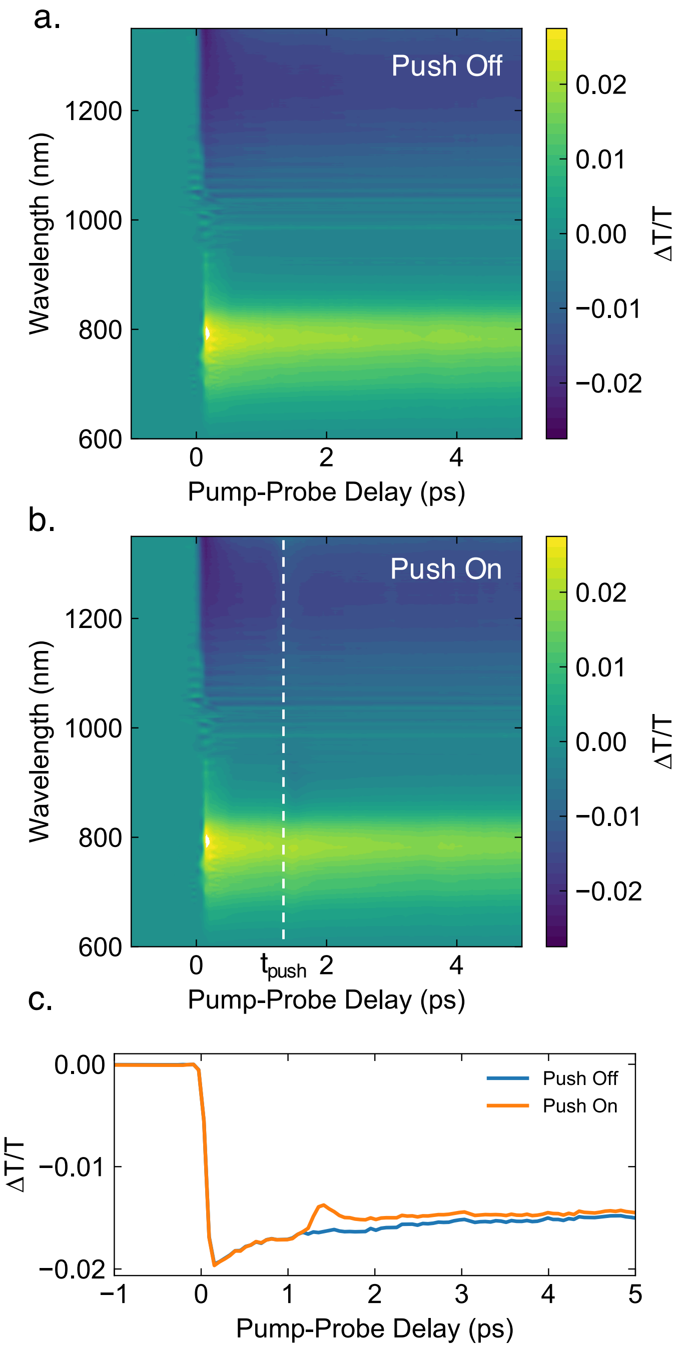

Supplementary Figure 2 | Pump-Probe (a) and Pump-Probe with Push (b) TA maps for a PIPCP:PCBM blend film. The pump-push delay is 1250 fs as indicated by the dotted line. (c) A comparison of the kinetics with and without push for a probe wavelength of 1200–1250 nm, and evidences the effect of the push pulse on the excited state population.

3. Push-Probe Transient Absorption for PIPCP:PCBM

Here are presented typical push-probe maps for two different cases. In the first case, the push-probe map is measured with a push fluence below the multiphoton excitation of the ground state threshold. In this case, the push-probe map is free from any transient absorption signal except for the coherent artefact around time zero. In the case of a push fluence above the multiphoton absorption threshold, we can observe in addition of the coherent artefact, positive time delays transient absorption signals that correspond to the ground state excitation of the sample, similar to the resonant pump-probe transient absorption. In this case, the two photon excitation does not populate the same excited states in the sample but leads within the time resolution of a few 100s of femtoseconds to similar transient absorption features.

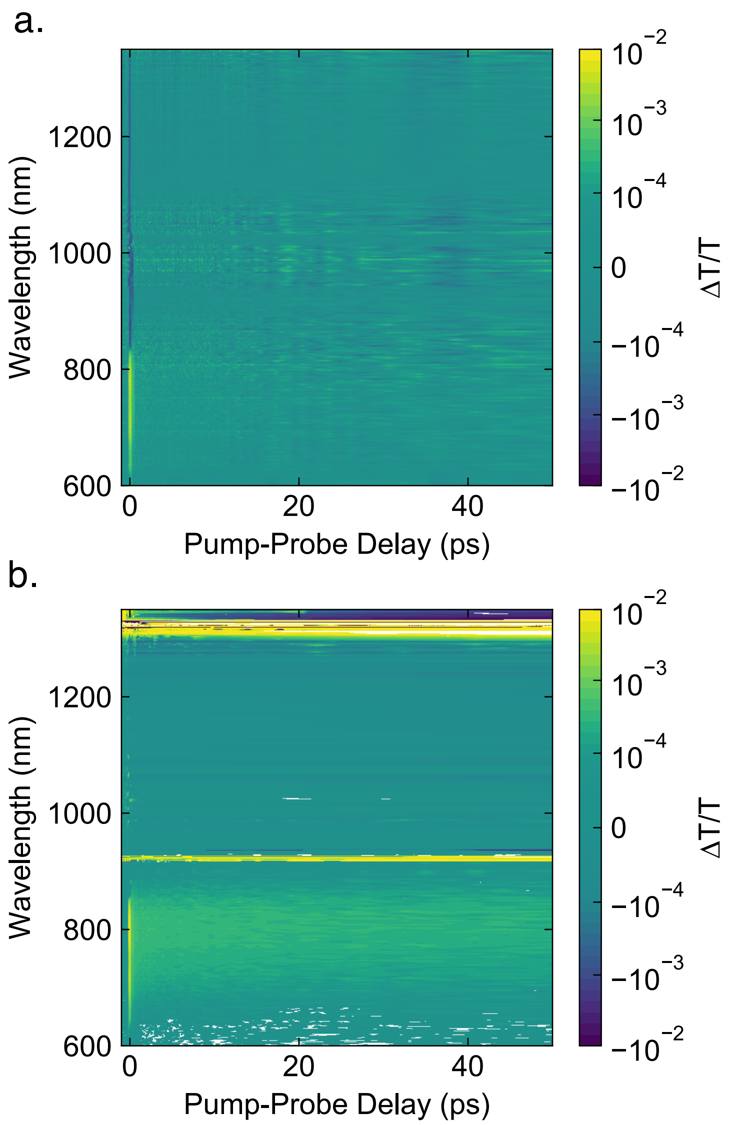

Supplementary Figure 3 | (a) Push-Probe TA map for a push fluence below multiphoton absorption threshold. Note the presence of the coherent artefact in the vicinity of time zero. (b) Push-Probe TA map for a push fluence above multiphoton absorption threshold. Note the similarity of the signal with the resonant Pump-Probe signals (GSB in the 650-800nm region) and the push scattering around 1350nm.

4. Confirmation of Electroabsorption Signature

Figure S4 shows the blend absorption for a PIPCP:PCBM blend as measured by photothermal deflection spectroscopy. Figure S4 also shows the electroabsorption (EA) from a working PIPCP:PCBM device. This spectrum is acquired by measured the relative change in reflection off the cathode (ΔR/R) at various wavelengths in response to a varying electric field applied across the electrodes of the device. The device was held under a small (-1 V) reverse bias to minimize charge injection from the electrodes. As can be seen from Fig. S4, the first derivative of the absorption matches the EA spectrum signalling that both of these measurements represent the bulk EA response. While both of these spectra are similar to that extracted from the push-induced EA (Fig. 4 in the manuscript), the push-induced EA represents the volume surrounding the donor-acceptor interface which need not be identical to the bulk volume. In this way, the push-induced serves as a local probe of blend morphology near the donor-acceptor interface where charge separation is occurring.

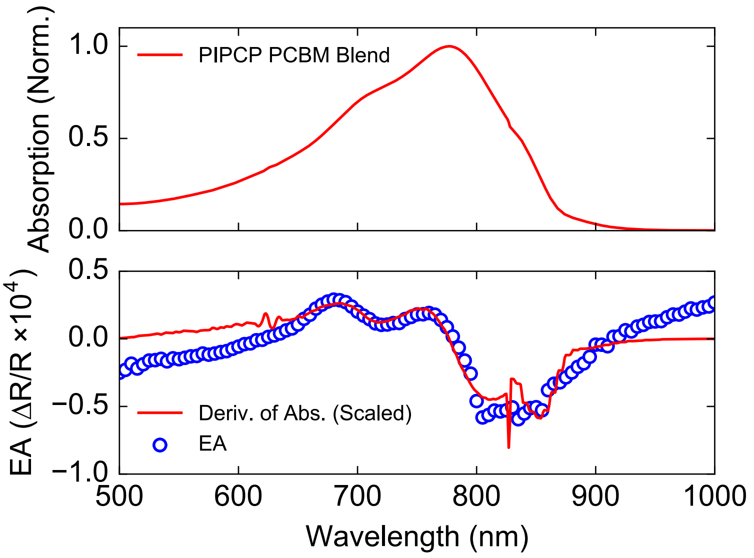

Supplementary Figure 4 | (top) blend absorption for a PIPCP:PCBM blend as measured by photothermal deflection spectroscopy. (bottom) Device-based electroabsorption (EA) spectrum as compared to the first derivative of the blend absorption.

5. Differential TA Maps

For t_{push}=1.4 ps, only charges are present in the blend. The short lifetime of the push-induced EA signals that these separated charges come back together rapidly, albeit in an altered configuration as compared to before the push-pulse arrived as indicated by the feature decaying on the 10 ps timescale. Eventually, however, the system can recover to the non-push-induced state as indicated by the zero push-induced, differential TA signal at 100 ps. In contrast, the push-induced, differential TA signal for t_{push}=0.4 ps does not fully recover and the long-lived (>100 ps) signal is attributed to an additional amount of charges present as indicated by the negative signals in the >1100nm region. For t_{push}=0.4 ps, singlet excitons have not been fully quenched to CT states, and, as a result, the push pulse centred at λ_{push}=1350 nm can interact with both unquenched singlet excitons as well as CT states and charges. The additional amount of long-lived charges only exhibited for short pump-push delays indicates that some singlet excitons may not be quenched to low-energy, tail states within the CT state density of states. Only with the additional energy supplied by the push pulse are they able to undergo full charge separation. This behaviour, however, is only representative of a small fraction of the initially excited excitons. By comparing the $\Delta T/T$ of the total charge population and the $\Delta(\Delta T/T)$ of additional charges created from the push pulse, we estimate that 99% of the initial exciton population is able to undergo efficient charge separation, confirming the basis for a high external quantum efficiency on the earliest of timescales.

For pump-push delays greater than 10 ps, a non-trivial amount of triplet excitons have been generated at the arrival of the push due to non-geminate charge recombination events. While push pulse interacting with the triplet does not result in EA, the push pulse moves the pushed system away from the distribution of excited states in the un-pushed system. If for instance, the push pulse excited the triplet exciton to a state that was subsequently and rapidly deactivated to the ground state, we would see a net reduction in triplet excitons until their

population starts to decay. In Fig S5, the negative signal appearing after 1 ps and peaked at λ=800 nm corresponds to a reduction in ground state bleach. From the shape of the corresponding bleach of photoinduced absorption gradually increasing beyond the spectral window of the measurement, we can assign this species to a reduction in triplet excitons. Consistent with the above analysis, the push pulse also reduces the number triplet excitons. This difference persists longer than the time limit of our setup (100 ps), but likely decays on the timescale of triplet excitons measured previously in PIPCP:PCBM blends. Importantly, we do not see long-lived (>100 ps) features in the PPP when using pump-push delays <10 ps when triplet excitons have not been appreciably formed.

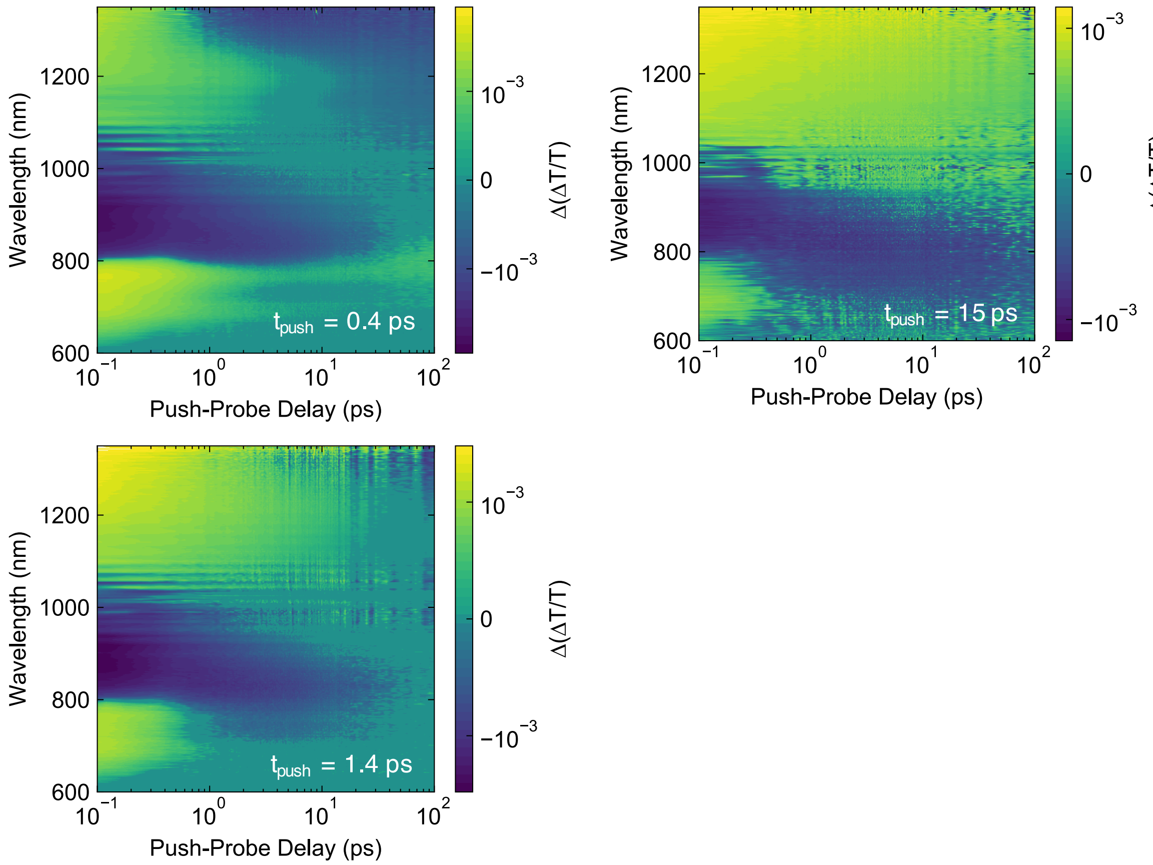

Supplementary Figure 5 | Push induced changes in TA spectra as a function of push-probe delay for various pump-push (t_{push}) delays.

6. Kinetics of Push Induced Differential TA

Figure S6 shows the kinetics of the EA signature (λ=800–850) and PIA bleach (λ=1200–1250) extracted from the spectra seen in Fig 3b of the manuscript. The PIA bleach is found to have a significantly shorter lifetime than the EA signature. This behaviour is expected. After the push pulse is absorbed by a CT state or free charge it is moved into a different electronic state with different PIA features, hence the bleach of the original PIA feature. Rapidly, this hot polaron thermalizes in place, reforming the original PIA spectrum and reducing the intensity of the bleach. However, the carrier is now an additional distance away from its original position and an EA signature remains due to the difference in the extent of the electric field generated by the electron-hole pair and the resulting photoinduced Stark effect upon neighbouring molecules.

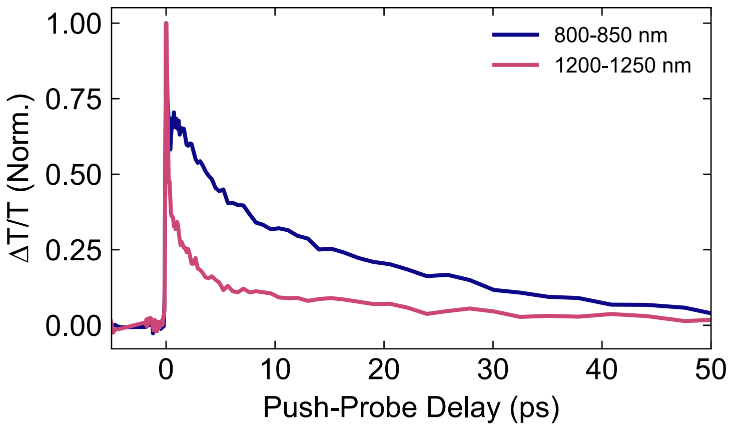

Supplementary Figure 6 | Push induced changes in TA kinetics for a pump-push (t_{push}) delay of 1.2 ps at wavelengths corresponding to the photoinduced electroabsorption (800–850) and the bleaching of CT states and free charges (1200–1250).

7. Model for Average Trap Spacing vs Urbach Energy

This section describes the approach we used to generate a simple model for how trap spacing evolves with Urbach energy as depicted in Fig. 6a in the manuscript. We start by taking the blend steady-state absorption spectrum for PIPCP:PCBM and identifying the energy below which the spectrum can be modelled with an exponential function (as is done to find the Urbach Energy). We can call this energy E_1. We can then generate a set of new absorption spectrums below this energy where we have systematically varied the Urbach energy (Fig. S8). By assuming that the absorption cross section is not strongly changing, we can use the absorption spectrum to approximate the shape of the density of states. To estimate the fraction of trap states we integrate from 0 eV to $E_1-0.1$ eV. Note that 0.1 eV is selected here to represent the energy at which CT states can no longer separate efficiently. To estimate the total number of states we can integrate from E_1 to 2 eV (over the main absorption band). A ratio of these two numbers gives an estimate for the fraction of trap states. We then multiply by the molecular density of the film to arrive at a density of trap states. Here we use 10^{27} m^{-3}. With the density known, we can now compute the average separation between trap sites by assuming a cubic lattice.

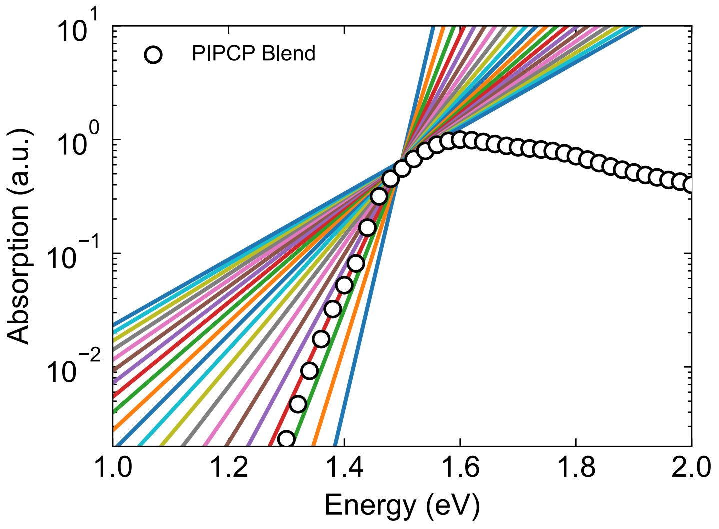

Supplementary Figure 7 | Absorption spectrum of a PIPCP:PCBM blend along with a set of hybrid absorption spectra where we have systematically varied the Urbach energy from 20 meV to 150 meV.

8. Internal Quantum Efficiency of PIPCP:PCBM

Figure S7 shows the internal quantum efficiency (IQE) of a PIPCP:PC_{61}BM device. Note that we have estimated the low-energy absorption (1.3-1.5 eV) of the active layer by back calculating the absorption spectrum from the electroluminescence spectrum of the device operating near open-circuit voltage, according to the relationship $A(E) \sim N(E)\ E^{-2}\ \exp(E/kT)$. Here, A(E) is the absorption at photon energy, E, N(E) is the number of photons emitted at energy E, k is the boltzmann constant, and T is the temperature. This is similar to the analysis shown in the manuscript by Vandewal *et al.*[2] As other estimates for absorption can include parasitic contributions from scattering and electrode absorption, this technique can provide higher accuracy at lower energies. The back-calculated absorption is then matched with the absorption spectrum measured by total reflectance in an integrating sphere. We show the curves for three separate devices.

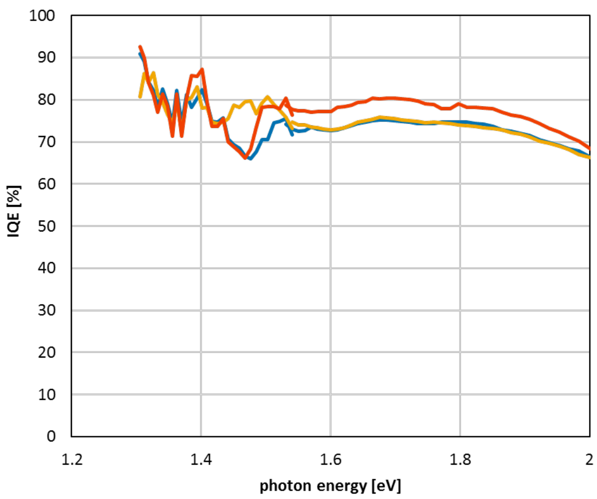

Supplementary Figure 8 | Internal quantum efficiency (IQE) versus photon energy for three identical PIPCP:PC_{61}BM devices.

Supplementary Note 1: Estimating Pump-Induced Electroabsorption

From the differential TA measured in the PPP configuration we directly measure the push-induced electroabsorption. In order to estimate the energy stored per charge pair, we need to convert the push-induced EA to pump-induced EA, a format that has been reported previously.[1] To do this we start by assuming that the charges are initially localised to the DA interface. This is consistent with the push induced EA signal (EA_{push}) being maximum for the shortest pump-push delay times. By subtracting EA_{push} at various time delays from the maximum push-induced EA (EA_{max}), we can estimate the pump-induced EA (EA_{pump}) as follows:

$$EA_{max} = EA[t]_{pump} + EA[t]_{push} \tag{1}$$

$$EA[0]_{pump} = 0 \quad and \quad EA_{max} = EA[0]_{push} \tag{2}$$

$$EA[t]_{pump} = EA_{max} - EA[t]_{push} \tag{3}$$

$$EA[t]_{pump} = EA[0]_{push} - EA[t]_{push} \tag{4}$$

In this formalism, each EA signal here should be treated with units of differential transmission. In our analysis, we do these subtractions on a per charge basis by dividing the measured EA signal by the bleach of the push-charges. To recover units of differential transmission we can multiply by the $\Delta T/T$ of the pushed charges.

Once we have the push-induced EA signal, we can move forward to estimate the energy stored in the electric field of the two separating charges according to the following equation:

$$Energy = EA[t]_{pump} * \frac{\varepsilon}{2Cen} \tag{5}$$

where ε is the dielectric constant, e is the charge of an electron and n is the density of pushed charges. C is a calibration factor between voltage and differential transmission extracted from quasi steady state measurements on a working device.

Supplementary Note 2: Description of the Pump-Push-Probe Transient Absorption Measurement

The Pump-Push-Probe experiment is based on an ultrafast broadband transient absorption described elsewhere. It allows the measurement of push induced change in transient absorption (TA) at 40 kHz with high time resolution in the visible to near infrared regions.

In brief, the probe pulse is a chirped white light continuum (500–1450nm) generated in YAG by focusing the fundamental of a PHAROS (Light Conversion) laser running at 40 kHz. After interaction with the sample, the probe beam is split with a low pass filter with 960 nm cut-off to obtain a transmitted near infrared beam and a reflected visible beam. The near infrared and visible probe beams are injected in a spectrometer with InGaAs and Si photodiode array detector, respectively. The two sensors are synchronized and read simultaneously at 40 kHz, allowing shot-to-shot detection.

The pump, probe and push pulses are spatially overlapped in the sample through a boxcar geometry (see Fig 3a in the manuscript), and temporally delayed using a piezoelectric delay stage for the pump-probe delay, and a DC servo stage for the pump-push delay.

The 750 nm, <12fs pump pulse is generated in a non-collinear optical parametric amplifier (NOPA) pumped with the second harmonic of the PHAROS laser (type II phase matching BBO). A chopper wheel allows us to build a sequence of probe pulses with and without a pump as described in Fig 3b in the manuscript. This allows for the calculation of the pump-induced change in transmission ($\Delta T/T$) for two consecutive probe pulses.

The 1350 nm, <300fs push pulse is generated in a commercial ORPHEUS (Light Conversion) OPA and chopped synchronously with the push chopper so as to generate the sequence of pulses shown in Fig 3b in the manuscript. This pulse sequence allows to calculate the classic pump induced change in transmission ($\Delta T/T$), as well as the pump induced change in transmission in the presence of the push, which allows to calculate the push induced change

in transient absorption (fig 3.b). The push induced change in transmission in the absence of pump (push TA) is also recorded and allows us check for multiphoton excitation of the ground state.

The latter signal allows us to set the maximum push fluence that can still be considered as a clean perturbation of the out of equilibrium, pump generated populations. The pump and push fluences are set so as to obtain a good signal to noise on the pump-push-probe signal $\Delta(\Delta T/T)$ with minimum pump and push fluences. This signal could be used as a correction to the signal for push fluences above the multiphoton ground state excitation, but at the detriment of the signal to noise. In the limit of a small pump excitation density (<10% of the molecules excited by the pump) the push multiphoton excitation of the ground state is not influenced by the presence of the pump in the sample (from the push multiphoton absorption perspective, the pump did not change much the ground state population). In that case, the Push-Probe data containing the transient absorption signatures of the push multiphoton excitation, can be subtracted directly from the Pump-Push data (corrected Pump-Push data = $DT/T_{[Pump\text{-}Push\text{-}Probe]}$ - $DT/T_{[Pump\text{-}Probe]}$ – $DT/T_{[Push\text{-}Probe]}$), to suppress the contribution from push multiphoton absorption, at the expense of doubling the noise level.

The pump-probe signal measured is used to check for sample degradation and for referencing the excited state populations at the push arrival time.

Supplementary References

1. Gelinas, S. *et al.* Ultrafast Long-Range Charge Separation in Organic Semiconductor Photovoltaic Diodes. *Science.* **343,** 512–516 (2014).
2. Vandewal, K. *et al.* Efficient charge generation by relaxed charge-transfer states at organic interfaces. *Nat Mater* **13,** 63–68 (2014).
